# How Emotion Regulation and Illness Identity Shape Mental Health in Adults with Congenital Heart Disease

**DOI:** 10.3390/medsci14010002

**Published:** 2025-12-19

**Authors:** Anna-Lena Ehmann, Daniel T. Marggrander, Janina Semmler, Felix Berger, Paul C. Helm, Constanze Pfitzer

**Affiliations:** 1 National Register for Congenital Heart Defects, Augustenburger Platz 1, 13353 Berlin, Germany; anna-lena.ehmann@charite.de (A.-L.E.); paul.helm@dhzc-charite.de (P.C.H.); 2 Deutsches Herzzentrum der Charité, Department of Congenital Heart Disease—Pediatric Cardiology, Augustenburger Platz 1, 13353 Berlin, Germany; 3 Charité—Universitätsmedizin Berlin, Corporate Member of Freie Universität Berlin and Humboldt-Universität zu Berlin, 10117 Berlin, Germany; 4 Department of Obstetrics, Charité—Universitätsmedizin Berlin, Corporate Member of Freie Universität Berlin and Humboldt-Universität zu Berlin, 13353 Berlin, Germany

**Keywords:** adult congenital heart disease, anxiety, depression, emotion regulation, illness identity, illness perceptions

## Abstract

**Background/Objectives:** Adults with congenital heart disease (ACHD) are at increased risk for mental health problems, particularly depression and anxiety. Emerging evidence suggests that psychological rather than purely medical factors may play a decisive role in explaining individual differences in emotional adjustment. However, comprehensive models integrating multiple cognitive and emotional domains remain scarce. This study aimed to identify the psychological variables most strongly associated with depressive and anxiety symptoms in ACHD when considered simultaneously to inform priorities for psychosocial interventions. **Methods:** A total of 1136 ACHD (aged 18–85 years; 59.7% female) from the National Register for Congenital Heart Defects, Berlin, completed an online survey assessing depression, anxiety, emotion regulation, illness perceptions, and illness identity. Correlational and multiple regression analyses were conducted, controlling for sociodemographic characteristics, CHD severity, and secondary diseases. Significance level for regression models was set at *p* < 0.025 due to Bonferroni correction. **Results:** Rumination showed the strongest positive correlations with both depression and anxiety, whereas acceptance was most negatively correlated. In multiple regression analyses, rumination (highest unique variance explanation with semi-partial R^2^ = 0.068 resp. 0.072) and illness engulfment emerged as the most strongly associated predictors of depressive and anxiety symptoms. Illness-related concerns were not significant predictors. **Conclusions:** The findings highlight the key role of repetitive negative thinking and an engulfed illness identity in the development of emotional distress among ACHD. Psychotherapeutic interventions targeting rumination, fostering psychological distance from illness identity, and promoting a multifaceted self-concept may be particularly beneficial in this population.

## 1. Introduction

With a prevalence of 0.8–1.2%, congenital heart disease (CHD) is the most common congenital malformation [1]. Thanks to medical advances, around 90% of those affected now reach adulthood [2,3,4,5]. As a result, the focus is now shifting from purely life-prolonging medical aims to quality of life and mental health issues of adults with CHD (ACHD) [6,7]. Numerous studies point to illness-related stress factors in connection with CHD and reveal an increased risk of mental illness in this patient group [7,8,9,10,11,12,13,14], whereby depression and anxiety disorders are considered the two most common ones in ACHD [8,9,15,16]. The exact processes and key variables involved in the development and maintenance of depressive and anxiety symptoms in ACHD are only partially investigated so far, leaving a significant research gap in this highly relevant and increasingly prominent topic.

The evidence on how medical characteristics relate to psychological outcomes in CHD remains inconsistent. While studies conducted in Germany generally fail to demonstrate a meaningful link between CHD severity and symptoms of anxiety or depression [17,18], findings from international cohorts paint a more mixed picture. Some of these investigations have observed associations between depressive or anxious symptomatology and the complexity of the cardiac defect, whereby these effects tend to diminish once multivariate models control for functional status, particularly New York Heart Association class [15,19,20,21]. Accordingly, medical variables such as CHD severity do not seem to play a central role in the context of psychopathological symptoms, so that cognitive and emotional processing operations are assumed as crucial components in the development of mental illnesses in ACHD. More precisely, individual perceptions of illness and illness identity constructs have been found to be correlated with psychological distress in ACHD so far [17,22,23,24,25,26]. Illness perceptions refer, for example, to the subjective sense of control, the emotional responses elicited by the illness, and the perceived benefits of medical treatment [27,28], whereas illness identity encompasses how a chronic illness becomes integrated into one’s identity and shapes self-perception [29]. A distinction is made between adaptive (acceptance and enrichment) and maladaptive (engulfment and rejection) identity dimensions [26,29,30], with more adaptive forms of identity being associated with lower psychological distress and lower utilisation of mental health treatment [24,25,26,31].

Furthermore, the use of individual emotion regulation strategies in everyday life has been identified in connection with mental health and life satisfaction in ACHD [32,33]. However, up to now most studies focus on illness-related constructs, and emotion regulation strategies in ACHD have been rather neglected to date, even though their central relevance across diverse mental illnesses as well as in the context of disease adaptation has been widely proven [34,35,36,37,38,39,40,41]. Specifically, emotional dysregulation such as difficulties with cognitive control, inhibition, or detaching from negative thoughts are considered core mechanisms in the development and maintenance of mental illnesses and particularly well documented in cases of depression [42,43]. A key influence, not only for depression but also anxiety, seems to be the frequent use of maladaptive strategies like rumination and the rare use of adaptive strategies such as reappraisal and redirecting attention to planning [43,44,45]. Accordingly, emotion regulation strategies have been revealed as an effective starting point in psychotherapeutic treatment, also for ACHD [33,46,47,48]. Emotion regulation also acted as a moderator between chronic stress and the risk of cardiovascular disease such as blood pressure [49], meaning that consideration of emotion regulation strategies in ACHD is relevant not only in terms of mental well-being but also with regard to potential physical risks.

Previous studies mainly examined the relationships between illness perceptions, illness identity or emotion regulation and psychological well-being in ACHD independently and comprehensive statistical models are still rare [22,25,32,50]. The present study addresses this issue by simultaneously recording illness perception, illness identity and emotion regulation strategies as potential predictors of symptoms of depression and anxiety in ACHD. Conceptually, the mentioned constructs may share common mechanisms related to the cognitive and emotional processing of illness, as all are involved in illness adjustment, suggesting partial theoretical overlap. Nevertheless, including all constructs in the analyses allows for a broader understanding of the factors primarily associated with depressive and anxiety symptoms, while accounting for overlapping effects or shared variance. This approach helps identify key targets for psychosocial interventions, given that mild depressive symptoms not only significantly diminish patients’ quality of life [51] but may also contribute to reduced adherence in cardiological follow-up settings [52].

## 2. Materials and Methods

### 2.1. National Register for Congenital Heart Defects

By October 2025, the National Register for Congenital Heart Defects (NRCHD) had developed into one of Europe’s largest and most detailed repositories of data on congenital heart disease. Containing medical records from roughly 60,000 individuals, the register serves as a valuable and reliable resource for advancing clinical research in this medical field [53]. Enrolment in the NRCHD is entirely voluntary and depends on patients providing broad informed consent. Through this authorization, participants permit the register to gather and maintain medical information from their healthcare providers, making it available for ongoing and future research projects. Moreover, participants may withdraw their consent at any time, ensuring full control over their personal data.

### 2.2. Study Design

In total, *N* = 1486 adults with congenital heart disease (ACHD) registered in the NRCHD took part in the web-based and cross-sectional survey. Eligibility for participation required being at least 18 years old, having a diagnosis of CHD, providing written informed consent, and possessing a valid email address. Individuals were excluded if they lacked sufficient German language proficiency or did not have internet access. Furthermore, participants were not considered if parents indicated they would complete the questionnaire on behalf of their child, for instance due to cognitive limitations. A total of 7692 individuals who fulfilled these conditions received an email invitation to take part in the study. Of those invited, 2154 (28.0%) initiated the survey, and 1486 respondents completed all survey sections, resulting in a final completion rate of 19.3%. For 223 participants, the available medical data were insufficient to determine CHD severity, and an additional 127 respondents did not report their net income. To maintain the clinical interpretability of the findings, the analyses were therefore conducted with a reduced sample of 1136 participants. No data imputation procedures were applied. The data collection process took place during the first quarter of 2024. Data were obtained through an online questionnaire, designed to explore various psychosocial dimensions among ACHD individuals. Participants were invited via email and asked to complete self-assessment surveys covering socio-demographic characteristics, illness-related beliefs and identity, as well as aspects of mental health and emotion regulation. In addition, medical data from the NRCHD registry were integrated into the statistical evaluation, with cardiac conditions categorized following the International Pediatric and Congenital Cardiac Code (IPCCC) classification system [54].

### 2.3. Measures

#### 2.3.1. Illness Perception

Perceptions of illness were assessed using the Brief Illness Perception Questionnaire (BIPQ) [27]. This instrument includes nine items designed to capture both cognitive and emotional aspects of how individuals perceive their illness. It addresses factors such as perceived control, treatment effectiveness, symptom burden, functional limitations, emotional involvement, disease-related worries, and understanding of the condition. Because CHD is a lifelong, inborn condition, the items concerning expected illness duration and assumed causes were excluded. Each statement is rated on a 0–10 scale, where 0 represents minimal agreement. Mean scores are calculated for each item to represent the overall perception profile.

#### 2.3.2. Illness Identity

To assess how individuals integrate their illness into their self-concept, the Illness Identity Questionnaire (IIQ) [29] was employed. The IIQ evaluates four dimensions of illness identity: engulfment, rejection, acceptance, and enrichment. The first two dimensions—engulfment and rejection—reflect maladaptive or insufficient integration, whereas acceptance and enrichment indicate adaptive incorporation of the illness into one’s identity. Each dimension is represented by several items rated on a five-point Likert scale (1 = very low to 5 = very high). Mean values are computed for each subscale to quantify the respective identity dimension.

#### 2.3.3. Mental Health

##### Depressive Symptoms

Depressive symptoms were assessed with the Patient Health Questionnaire-9 (PHQ-9) [55], which comprises nine questions addressing how frequently specific depressive symptoms occurred over the previous two weeks. Each item is rated on a four-point scale, and total scores range from 0 to 27. Depending on the overall score, depressive severity is classified as mild (5–9), moderate (10–14), moderately severe (15–19), or severe (20–27).

##### Anxiety Symptoms

Anxiety symptoms were measured using the Generalized Anxiety Disorder Scale (GAD-7) [56]. This questionnaire includes seven items assessing the degree to which anxiety symptoms interfere with daily functioning. Each item is scored on a four-point scale, and the total score (0–21) reflects the intensity of anxiety. Severity levels are interpreted as mild (5 points), moderate (10 points), and severe (15 points).

#### 2.3.4. Emotion Regulation

Emotion regulation strategies were examined using the Heidelberg Form for Emotion Regulation Strategies (H-FERST) [57]. This tool consists of 28 items measuring the use of eight distinct regulation strategies: reappraisal, problem-solving, acceptance, social sharing, rumination, avoidance, expressive suppression, and suppression of experience. Participants rate how often they use each strategy on a five-point scale (1 = never, 5 = always).

#### 2.3.5. Sociodemographic and Medical Variables

Sociodemographic information collected included sex, age, education level, employment, relationship status (firm relationship yes/no), city size and net monthly income (low: <1750 €, medium: 1750 €–3999 € and high: >3999 €). In this study, ‘education’ refers exclusively to participants’ highest level of completed formal schooling, while ‘pupil’ implicates the person is still in school and does not yet have a completed graduation or certificate. In contrast, ‘employment’ captures participants’ current occupational status at the time of the survey. Because individuals may simultaneously engage in more than one activity, the ‘employment’ section allowed multiple responses. For example, some participants were still attending school or university (‘school’ or ‘university’ as an employment category) while also working a part-time job. This structure was chosen to accurately reflect the diverse and often overlapping educational and occupational situations within the ACHD population. Moreover, CHD severity (simple/moderate/complex) according to Warnes et al. [58] and the presence of secondary diseases (yes/no) were recorded.

### 2.4. Reliability of Measures

Cronbach’s alpha was calculated to assess the internal consistency of all scales used in the study. For the IPQ, Cronbach’s alpha was 0.62. However, it should be noted that the IPQ is not intended as a unidimensional summative scale, but rather as a measure of individual items assessing different illness perceptions. The IIQ subscales demonstrated high internal consistency, with α = 0.92 for engulfment, 0.81 for rejection, 0.80 for acceptance, and 0.93 for enrichment. Measures of depressive and anxiety symptoms also showed good to excellent reliability: HADS-D α = 0.82, HADS-A α = 0.81, PHQ-9 α = 0.87, and GAD-7 α = 0.89. The H-FERST subscales, assessing emotion regulation strategies, yielded acceptable to good reliability across strategies, with α ranging from 0.78 to 0.85.

### 2.5. Statistical Analyses

Data analysis was conducted using SPSS (Version 29.0) and RStudio (Version 4.3.3). Pearson’s bivariate correlations were calculated in order to assess relations between all psychological constructs. Two distinct multiple regression models were calculated with symptoms of depression and anxiety each as dependent variables. Illness perceptions, illness identity dimensions and emotion regulation strategies were included as independent variables in both regression models. Since various sociodemographic variables are linked to mental health in ACHD [8], all above mentioned socio-demographic variables as well as CHD severity and presence of secondary diseases were included as covariates. To control for multiple comparisons across the two regression models, a Bonferroni correction was applied at the level of the models. Accordingly, the significance threshold was adjusted to α = 0.025 for each model. Assumption checks were conducted for both regression models (PHQ-9 and GAD-7). Linearity between the predictors and the dependent variables was evaluated using partial regression plots. All plots showed approximately linear relationships without systematic curvature or other non-linear patterns, indicating that the linearity assumption was fulfilled in both models. The normality of residuals was assessed using P–P plots. In each model, the standardised residuals closely followed the diagonal line, indicating an overall satisfactory approximation to a normal distribution. Autocorrelation of residuals was examined using the Durbin–Watson statistic. The values were 2.010 (PHQ-9) and 2.009 (GAD-7), suggesting no residual autocorrelation in either model. Influential observations were identified through Cook’s distance and leverage values. In both models, some cases exhibited elevated leverage (maximum = 0.105), exceeding the theoretical threshold for 35 predictors (2·p)/*N* = 0.062) according to Igo (2010; [59]), whereby Huber (1981; [60]) suggests a cutoff value of 0.2. As Cook’s distance values remained consistently low (maximum = 0.032 for PHQ-9; 0.029 for GAD-7) and far below the conventional cut-off of 1, all observations were retained in both analyses. Since residual plots indicated heteroscedasticity, parameters were calculated using robust standard errors according to the HC4 method. Variance inflation factors (VIF) of both regression models ranged between 1.113 and 3.923, indicating no problematic multicollinearity considering our previously defined critical cut-off of 5.

Blockwise inclusion methods, together with the calculation of semi-partial R^2^ in a regression model including all predictors simultaneously, were used to examine the unique and incremental variance explained by the significant variables. First, all predictors were entered simultaneously in a single regression model to estimate the semi-partial R^2^ of each predictor, reflecting their unique contribution to the outcome variable. Second, a hierarchical (blockwise) regression was conducted to evaluate the variance explained by conceptually ordered predictor sets: sociodemographic and medical control variables were entered in the first step, illness perceptions in the second step, illness identity in the third step, and emotion regulation variables in the fourth and final step. This model allowed the assessment of the incremental R^2^ associated with each block. Third, additional hierarchical regression analyses were performed in which each predictor block was entered last once, thereby enabling a direct comparison of the incremental variance explained by each block when controlling for all other predictor sets. All predictors were entered regardless of their statistical significance or the amount of variance explained by any individual block.

In order to examine gender differences in the predictors of psychopathological symptoms, separate regression models were additionally calculated for men and women.

### 2.6. Ethical Statement

The study received ethical approval from the Charité Ethics Committee, confirming its compliance with research and data protection standards (EA4/178/22, 24 October 2022). All participants provided written informed consent prior to their inclusion in the study.

## 3. Results

### 3.1. Study Cohort

The study cohort comprised N = 1136 ACHD, aged 18–85 years (M = 36.73, SD = 14.03). The CHD severity was classified as simple in 142 participants (12.5%), moderate in 618 (54.4%), and complex in 376 (33.1%) patients. The sample included 458 males (40.3%) and 678 females (59.7%). Table 1 shows sociodemographic characteristics of the sample.

### 3.2. Illness-Related Constructs, Emotion Regulation and Psychopathological Symptoms

Depressive symptoms were significantly associated with all aspects of illness perception, illness identity and emotion regulation (all *p* < 0.001). The strongest positive correlation was observed with the emotion regulation strategy rumination (*r* = 0.486), while the strongest negative correlation was found with acceptance (*r* = −0.422). Correlations with illness perception facets ranged between *r* = −0.171 (perceived treatment benefit and understanding of the CHD) and *r* = 0.353 (emotional impact of CHD). Correlations with illness identity dimensions ranged between *r* = −0.219 (acceptance) and *r* = 0.404 (engulfment).

Anxiety symptoms were significantly correlated with all aspects of illness identity, illness perception and emotion regulation besides suppression of experience (*r* = 0.056, *p* = 0.061). The strongest positive correlation was found between anxiety symptoms and rumination (*r* = 0.504, *p* < 0.001), whereas the highest negative correlation was observed between anxiety symptoms and acceptance (*r* = −0.467, *p* < 0.001). Correlations with illness perception facets ranged between *r* = −0.131 (treatment benefit) and *r* = 0.382 (emotional impact of CHD). Correlations with illness identity dimensions ranged between *r* = −0.215 (acceptance) and *r* = 0.398 (engulfment).

Depressive and anxiety symptoms showed a correlation of *r* = 0.803 (*p* < 0.001). Correlations between all psychological constructs are displayed in Table 2.

### 3.3. Predictors of Depressive Symptoms

Multiple linear regression analysis revealed perceived symptom complaints (*ß* = 0.120, *p* = 0.006]), engulfment (*ß* = 0.247, *p* < 0.001), enrichment (*ß* = −0.065, *p* = 0.019), and the emotion regulation strategies rumination (*ß* = 0.321, *p* < 0.001), reappraisal (*ß* = −0.149, *p* < 0.001), acceptance (*ß* = −0.137, *p* < 0.001) and suppression of expression (*ß* = 0.098, *p* = 0.003) as significant predictors of depressive symptoms, even when controlling for sex, age, CHD severity, education level, relationship status, city size, net income and the presence of secondary diseases. Of the sociodemographic and medical variables, only CHD severity (reference: CHD complex; CHD simple: *ß* = 0.081, *p* = 0.002, CHD moderate: *ß* = 0.083, *p* = 0.001) showed significant effects. The regression model showed a variance explanation of R^2^ = 0.465 (corrected R^2^ = 0.448) with a significant change in F (*p* < 0.001).

**Figure 1:**
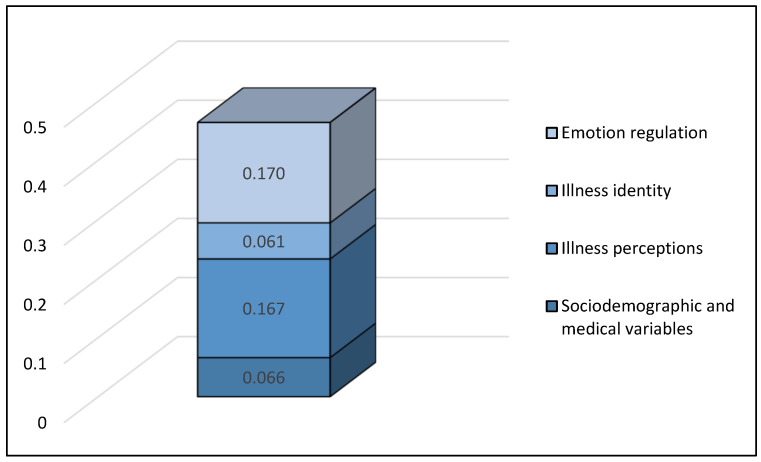
Additional explained variance (ΔR^2^) of each construct family in predicting depressive symptoms (PHQ-9) when conducting a blockwise regression (method: inclusion, order: Sociodemographic and medical variables, illness perceptions, illness identity, emotion regulation).

Figure 1 visualizes the changes in R^2^ for each construct family when all predictors were taken into account in a blockwise regression model in the following order: Sociodemographic and medical variables (R^2^ = 0.066), illness perceptions (R^2^ = 0.167), illness identity (R^2^ = 0.061), and emotion regulation (R^2^ = 0.170).

**Table 2:** Pearson’s bivariate correlations between psychological constructs.

	1	2	3	4	5	6	7	8	9	10	11	12	13	14	15	16	17	18	19	20
Illness Perceptions (IPQ)																				
(1) Impairment																				
(2) Control	−0.07 *																			
(3) Treatment benefit	0.07 *	0.11 ***																		
(4) Perceived symptoms	0.82 ***	−0.06 *	0.02																	
(5) Worries	0.60 ***	−0.16 ***	0.08 **	0.61 ***																
(6) Understanding	−0.06	0.18 ***	0.05	−0.03	−0.13 ***															
(7) Emotional involvement	0.59 ***	−0.11 ***	0.05	0.56 ***	0.72 ***	−0.08 **														
Illness Identity (IIQ)																				
(8) Engulfment	0.76 ***	−0.12 ***	0.03	0.72 ***	0.70 ***	−0.11 ***	0.72 ***													
(9) Rejection	0.13 ***	−0.07 *	−0.13 ***	0.14 ***	0.18 ***	−0.24 ***	0.24 ***	0.25 ***												
(10) Acceptance	−0.13 ***	0.03	0.23 ***	−0.12 ***	−0.22 ***	0.19 ***	−0.25 ***	−0.27 ***	−0.46 ***											
(11) Enrichment	0.22 ***	0.11 ***	0.23 ***	0.19 ***	0.14 ***	0.19 ***	0.18 ***	0.19 ***	−0.13 ***	0.35 ***										
Emotion Regulation (HFERST)																				
(12) Rumination	0.11 ***	−0.07 *	0.06 *	0.12 ***	0.22 ***	−0.06 *	0.26 ***	0.24 ***	0.00	−0.06 *	0.02									
(13) Reappraisal	−0.10 ***	0.13 ***	0.16 ***	−0.08 **	−0.11 ***	0.12 ***	−0.14 ***	−0.13 ***	−0.12 ***	0.21 ***	0.35 ***	−0.10 ***								
(14) Acceptance	−0.17 ***	0.10 **	0.10 **	−0.15 ***	−0.23 ***	0.17 ***	−0.31 ***	−0.25 ***	−0.19 ***	0.30 ***	0.23 ***	−0.33 ***	0.43 ***							
(15) Problem solving	−0.20 ***	0.09 **	0.09 **	−0.17 ***	−0.15 ***	0.20 ***	−0.16 ***	−0.20 ***	−0.19 ***	0.23 ***	0.19 ***	0.08 **	0.36 ***	0.39 ***						
(16) Suppression of expression	−0.01	0.01	−0.04	0.04	0.01	−0.01	0.02	0.04	0.19 ***	−0.09 **	−0.06	0.12 ***	0.02	0.04	0.16 ***					
(17) Suppression of experience	−0.04	0.00	−0.05	−0.03	−0.06 *	−0.05	−0.06 *	0.00	0.22 ***	−0.11 ***	−0.06 *	−0.06 *	0.04	0.06	0.06 *	0.59 ***				
(18) Avoidance	0.08 *	−0.01	−0.04	0.08 *	0.11 ***	−0.09 **	0.16 ***	0.14 ***	0.23 ***	−0.13 ***	−0.06	0.19 ***	−0.09 **	−0.22 ***	−0.05	0.26 ***	0.41 ***			
(19) Social sharing	−0.02	0.06 *	0.10 ***	−0.03	−0.04	0.08 **	−0.02	−0.06 *	−0.23 ***	0.14 ***	0.20 ***	0.04	0.24 ***	0.06 *	0.16 ***	−0.43 ***	−0.38 ***	−0.16 ***		
(20) GAD-7	0.24 ***	−0.11 ***	−0.13 ***	0.27 ***	0.31 ***	−0.12 ***	0.38 ***	0.40 ***	0.16 ***	−0.22 ***	−0.10 **	0.50 ***	−0.33 ***	−0.47 ***	−0.15 ***	0.14 ***	0.06	0.20 ***	−0.12 ***	
(21) PHQ-9	0.27 ***	−0.13 ***	−0.14 ***	0.30 ***	0.30 ***	−0.14 ***	0.35 ***	0.40 ***	0.16 ***	−0.22 ***	−0.12 ***	0.49 ***	−0.33 ***	−0.42 ***	−0.17 ***	0.20 ***	0.09 **	0.21 ***	−0.17 ***	0.80 ***

Note, Blue colouring indicates a negative correlation, whereas red colouring indicates a positive correlation. The stronger the colour, the stronger the correlation. * *p* < 0.05, ** *p*< 0.01, *** *p* < 0.001 (two-tailed significance). *N* = 1136.

In order to examine the incremental variance of the four constructs in depressive symptoms, blockwise inclusions were calculated. Emotion regulation strategies showed the biggest incremental variance when added last (R^2^ = 0.170). The incremental variance explained by illness identity beyond the three constructs was R^2^ = 0.018, while sociodemographic and medical factors accounted for an additional R^2^ = 0.016, and illness perception contributed an incremental R^2^ = 0.009.

The comparison of the two regression models for men and women revealed that, for men, the significant predictors were engulfment (*ß* = 0.201, *p* = 0.012), rumination (*ß* = 0.367, *p* < 0.001), reappraisal (*ß* = −0.134, *p* = 0.007), acceptance (*ß* = −0.112, *p* = 0.023), and suppression of expression (*ß* = 0.117, *p* = 0.020). For women, significant predictors included CHD severity (Reference: complex; low: *ß* = 0.149, *p* < 0.001, moderate: *ß* = 0.120, *p* < 0.001), the presence of secondary diseases (*ß* = 0.076, *p* = 0.010), engulfment (*ß* = 0.282, *p* < 0.001), rumination (*ß* = 0.285, *p* < 0.001), reappraisal (*ß* = −0.148, *p* < 0.001), and acceptance (*ß* = −0.149, *p* < 0.001).

### 3.4. Predictors of Anxiety Symptoms

Multiple linear regression analysis revealed emotional concern (*ß* = 0.112, *p* = 0.011), engulfment (*ß* = 0.231, *p* < 0.001) and the emotion regulation strategies rumination (*ß* = 0.330, *p* < 0.001), reappraisal (*ß* = −0.158, *p* < 0.001) and acceptance (*ß* = −0.199, *p* < 0.001) as significant predictors of anxiety symptoms, when controlling for sex, age, CHD severity, education level, relationship status, city size, net income and the presence of secondary diseases. Similarly to depressive symptoms, only CHD severity (reference: CHD complex; CHD simple: *ß* = 0.065, *p* = 0.017, CHD moderate: *ß* = 0.059, *p* = 0.023) showed significant effects considering medical and sociodemographic variables. The regression model showed a variance explanation of R^2^ = 0.469 (corrected R^2^ = 0.452) with a significant change in F (*p* < 0.001).

**Figure 2:**
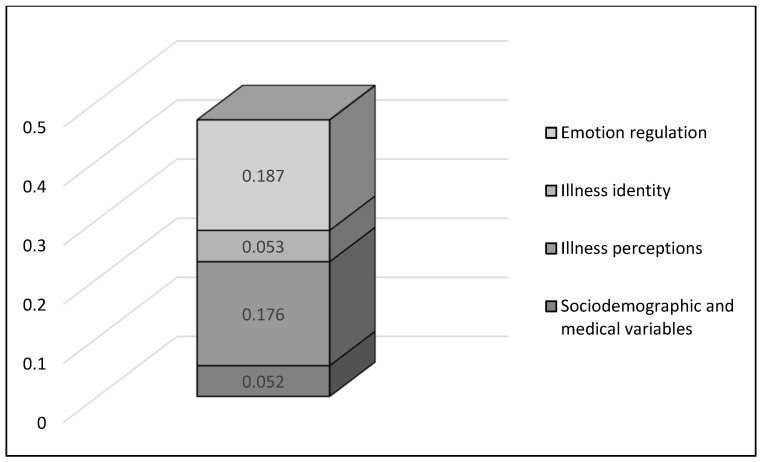
Additional explained variance (ΔR^2^) of each construct family in predicting anxiety symptoms (GAD-7) when conducting a blockwise regression (method: inclusion, order: Sociodemographic and medical variables, illness perceptions, illness identity, emotion regulation).

Figure 2 visualizes the changes in R^2^ for each construct family when all predictors were taken into account in a blockwise regression model in the following order: Sociodemographic and medical variables (R^2^ = 0.052), illness perceptions (R^2^ = 0.176), illness identity (R^2^ = 0.053), emotion regulation (R^2^ = 0.187).

To examine the incremental variance of the four constructs in anxiety symptoms, blockwise inclusions were calculated. Emotion regulation strategies again showed the largest incremental variance when added last (R^2^ = 0.187). Beyond the other three constructs, illness identity explained an additional R^2^ = 0.014, sociodemographic and medical factors contributed R^2^ = 0.012, and illness perception accounted for R^2^ = 0.009.

Table 3 and Table 4 present an overview of the significant predictors of the regression analyses. The comprehensive results of the regression analyses can be found in Tables S1 and S2 in the Supplementary Material.

Comparison of the regression models for men and women indicated that, in men, the significant predictors were engulfment (*ß* = 0.349, *p* < 0.001), rumination (*ß* = 0.397, *p* < 0.001), reappraisal (*ß* = −0.147, *p* = 0.003), and acceptance (*ß* = −0.137, *p* = 0.006). In women, significant predictors comprised simple CHD severity (Reference: complex; *ß* = 0.100, *p* = 0.008), being in school (*ß* = 0.052, *p* = 0.017), emotional involvement due to CHD (*ß* = 0.180, *p* = < 0.001), engulfment (*ß* = 0.171, *p* = 0.004), rumination (*ß* = 0.288, *p* < 0.001), reappraisal (*ß* = −0.151, *p* < 0.001), and acceptance (*ß* = −0.222, *p* < 0.001). The gender-specific regression models for depressive (a) and anxiety (b) symptoms are displayed in Tables S1a,b and S2a,b in the Supplementary Material.

## 4. Discussion

This study analysed predictors of depressive and anxiety symptoms in ACHD, considering illness perceptions, illness identity and emotion regulation strategies in multiple regression models. Since previous studies have mostly examined psychological constructs individually, the results of this study contribute to a more comprehensive overall picture of psychopathological correlates in ACHD.

Across all psychological constructs, correlations revealed the strongest positive associations between rumination and anxiety or depressive symptoms, while the strongest negative correlation was found for acceptance.

Multiple regression models still revealed rumination as the predictor most strongly associated with depressive and anxiety symptoms, while the illness identity dimension engulfment showed the second most pronounced relationship. Both variables were positively associated with psychopathological symptoms, indicating that frequent rumination and a strong feeling of being engulfed by the CHD are associated with higher mental burden. Further significant predictors of stronger depressive symptoms were more perceived symptom complaints and suppression of expression, while a stronger feeling of being enriched by the CHD, and a more frequent use of reappraisal and acceptance were associated with less depressive symptoms. Besides rumination and engulfment, further significant predictors of anxiety symptoms were, analogue to depressive symptoms, the strategies reappraisal and acceptance. Beyond, a stronger emotional concern through the CHD was associated with higher levels of anxiety.

Depressive and anxiety symptoms showed a high correlation with each other, indicating a substantial overlap in these symptom domains and suggesting a high likelihood of comorbidity. This result highlights the relevance of screening for both simultaneously and considering transdiagnostic mechanisms in psychosocial interventions.

Both, at the correlative level and in the regressions, the results indicate that emotion regulation strategies used in everyday life are superior to the illness-related constructs of illness identity and illness perceptions even when controlling for several co-variates in predicting depressive and anxiety symptoms. This is also supported by the semi-partial R^2^, which indicates that rumination is particularly relevant in practice for depressive symptoms, whereas the practical relevance of reappraisal is nearly equivalent to that of engulfment. For anxiety symptoms, it even appears that the practical relevance of reappraisal and acceptance exceeds that of engulfment. These results suggest that interventions for ACHD should not only focus on the perception of CHD and associated identity concepts but should also specifically develop broad and everyday emotion regulation skills.

Considering gender specific regression models, the present findings highlight rumination and feelings of engulfment as the strongest predictors of depressive and anxiety symptoms in both men and women, indicating that interventions targeting these cognitive-emotional processes may be particularly effective. Adaptive strategies, such as reappraisal and acceptance, showed protective effects across genders, with acceptance showing a negative association with anxiety especially in women. Additionally, disease-related factors, including CHD severity, secondary illnesses, and emotional involvement, seem to be more influential in women, emphasizing the need for gender-sensitive and individualized psychological interventions in patients with CHD.

While previous studies have found a particular strong correlation between life satisfaction and reappraisal [32], the present results emphasize the role of rumination and its association with depression and anxiety symptoms which becomes more apparent when disease-related constructs are taken into account. The strong correlation between rumination or engulfment and depressive and anxiety symptoms suggests that the dominating perception of unpleasant thoughts or feelings in daily life plays a particularly central role in relation to psychopathological symptoms. Our results therefore accompany a broad scientific consensus that maladaptive cognitive emotion regulation strategies, rumination in particular, play a central role in developing, maintaining and increasing depressive symptoms in both men and women and are associated with recurrence [42,43,61,62]. Accordingly, studies suggest that rumination can even be seen as a warning signal for a future depressive episode [63].

Particular attention should be paid to indications that rumination is also associated with increased suicidal thoughts [64]. At the same time, repetitive worries and rumination have previously been identified as typical features of anxiety disorders [44,65].

With regard to other emotion regulation strategies, more frequent use of reappraisal and acceptance was also associated with fewer symptoms of anxiety and depression, and should be considered in interventions alongside addressing rumination. Presumably due to its conceptual proximity to strategy reappraisal, the illness identity dimension enrichment proved to be a significant predictor, but only for depressive symptoms. Although the low effect size for enrichment should be taken into account, ACHD with depressive symptoms might benefit from working on positive evaluation patterns and the critical analyzation of negative thoughts within the scope of interventions. Moreover, suppression of emotional expression was associated with higher levels of depressive symptoms, albeit with a small effect size, suggesting that psychotherapeutic interventions should specifically encourage and support the expression of experienced emotions.

Overall, studies in the field of emotion regulation suggest that frequent use of maladaptive strategies plays a greater role in the development of psychopathology than infrequent use of adaptive strategies [45,66]. This is also reflected in the effect sizes of our results, where rumination shows a stronger effect than reappraisal and acceptance.

Interestingly, worries about CHD were not significantly associated with either anxiety or depression symptoms. This leads to the assumption that concerns about the illness, which may be more reality-oriented and focused on a specific topic, namely the illness, are less relevant to the development of psychopathological symptoms. Perhaps it is not primarily the content of the thoughts that matters, but rather the repetitive nature of rumination and the feeling of being consumed by the illness, which may be perceived as more stressful and burdensome. It may also be important to distinguish whether worries focus on the future course of the illness or on past experiences. Notably, in the present study, anxiety symptoms were measured as generalized anxiety, which is characterized by unspecific and broad concerns [67]. This may help explain the lack of a significant predictive effect of disease-specific worries. However, the insignificance of worries should be interpreted with caution, as they were only measured using a single item. Further studies should use other measurement instruments to gain a more accurate understanding of cognitive processes related to disease-related worries.

The perception of disease-related symptoms was associated with more depressive but not with anxiety symptoms. One possible explanation for this finding is that affected individuals may not perceive the symptoms as threatening. Since it is a congenital condition, the symptoms are familiar and can be anticipated, and thus may not be linked to anxiety symptoms. Nevertheless, the symptoms might be perceived as limiting, burdening or frustrating, which might be stronger associated with depression than with anxiety.

Feeling emotionally impaired by the CHD significantly predicted anxiety but not depressive symptoms. Instability and variability of emotional states has been found as characteristics for both depression and anxiety disorders in psychiatric samples [68]. Since the nature of the emotional impact (e.g., angry, sad, annoyed) was not specifically recorded, our findings do not allow for any precise explanations in this regard. However, this association of disease-related negative emotions with anxiety together with the non-significance of illness-related worries and the significant effect of engulfment suggests that it is not the mere mental engagement with the illness, but rather the emotional engulfment, becoming emotionally preoccupied by the disease and identification with it, that contribute to psychological distress in ACHD. As the perception of disease related symptoms only predicted symptoms of depression but not anxiety this additionally points to a distinction between the mere perception of physical limitations and the emotional occupation through the CHD. If, in addition to the physical symptoms, CHD is also perceived to have a significant impact on emotional well-being, which may lead to an increased perception of the threat posed by the disease.

Contrary to previous studies [17,18], CHD severity was found to be a significant predictor of anxiety and depressive symptoms in our results. However, studies have shown that this effect disappears when the New York Heart Association class is taken into account [15,19,20,21]. The results of this study and previous research generally support the hypothesis that the mere presence of a serious chronic illness is not automatically associated with psychopathological symptoms.

Overall, studies suggest that depression can develop from an anxiety disorder, and that anxiety disorders share broad similarities with affective disorders [65,67,69], as both tend to be internalising psychopathologies [70]. Therefore, these symptom patterns should not be considered in isolation, but rather as a common comorbidity in ACHD. This notion is further supported by the high correlation between anxiety and depressive symptoms observed in the present study. Accordingly, rumination and illness engulfment can be viewed as central transdiagnostic processes affecting both depressive and anxiety symptoms. This aligns with evidence that stable psychological dispositions, such as attachment styles and defense mechanisms, contribute to distress: insecure attachment increases distress via immature and neurotic defenses [71], while key defenses like self-assertion and passive aggression are central in networks of depressive and anxiety symptoms [72]. These findings suggest that targeting transdiagnostic processes and central defensive patterns may offer promising avenues for intervention across disorders.

### 4.1. Clinical Implications

Our study provides significant findings for the treatment and therapy of ACHD with psychological distress. The results underscore the need to consider individual emotion regulation strategies, especially rumination but further acceptance and reappraisal, in the treatment of ACHD and to pay particular attention to an engulfing illness identity. Although no causal conclusions can be drawn, the strong associations suggest that especially rumination and engulfment may serve as high-value targets for routine psychosocial screening in cardiology and ACHD care settings. Brief self-report tools including repetitive negative thinking and illness-related identity fusion such as screenings for depressive symptoms (e.g., Becks-Depression-Inventory or PHQ-9 [73]) or illness identity (e.g., IIQ [29]) could be feasibly integrated into standard appointments to identify individuals at heightened psychological risk.

From an interventional perspective, several psychotherapeutic components appear sufficiently established for trial readiness. Digital emotion regulation training based on cognitive-behavioural therapy (CBT) has been shown to reduce depressive and anxiety symptoms, as well as stress, in ACHD [33]. Similarly, mindfulness-based CBT interventions have demonstrated significant effects on depressive rumination [74,75,76]. In concrete, elements of CBT that specifically address repetitive negative thinking, such as metacognitive techniques, cognitive restructuring modules focused on disengaging from ruminative loops, and behavioural activation, could be transferred to the ACHD population. Likewise, interventions aimed at reducing illness engulfment may benefit from identity-focused work, including values clarification, self-complexity enhancement, and exercises that support differentiation between the self and the illness. These components can be delivered through digital, hybrid, or brief face-to-face formats, which facilitates integration into cardiology follow-up care. Future clinical trials should further examine the acceptability, feasibility, and effectiveness of such targeted interventions in ACHD to determine their potential role in routine psychosocial support.

### 4.2. Strengths and Limitations

Although this study offers highly relevant insights into emotional and cognitive processes associated with depressive and anxiety symptoms in ACHD in Germany, several limitations should be acknowledged. The cross-sectional design prevents causal conclusions and longitudinal studies are required to clarify temporal patterns in the development and maintenance of psychopathological symptoms. However, since current research in the field of emotion regulation assumes that emotion regulation strategies contribute to both the development and maintenance of mental illness [43], the identified strategies and identity concepts can be assigned a central role, independently of the exact causal mechanisms. Data were self-reported, introducing potential recall or social desirability biases. A substantial cohort of ACHD patients representing a wide range of disease severity was recruited, which can be regarded as a key strength of this study. Since the sample originated from the NRCHD, generalisability to ACHD populations outside Germany can be questioned. However, the NRCHD is considered representative of Europe, so the data set can be regarded as robust [53]. Future research might consider broader recruitment approaches, such as telephone or in-person enrolment, to reach participants without stable internet access, and include diverse clinical settings to enhance representativeness. Finally, since data were collected only in early 2024, the results may not capture later developments in mental health. To account for multiple testing, a Bonferroni correction was applied, indicating that the observed effects can be considered reasonably robust. Our study relied on screening instruments capturing symptoms of depression and anxiety. Consequently, while the findings provide valuable insights into symptom-level psychological patterns, they cannot yet be generalized to clinically confirmed disorders. Future research should therefore aim to examine the identified cognitive-emotional strategies in relation to formal ICD-based diagnoses to validate and extend these results. The PHQ-9 and GAD-7 could have been analyzed using ordinal regression based on severity categories. However, categorizing continuous scores via cut-offs reduces information, lowers statistical power, and may obscure subtle associations. Moreover, our recent study showed that severity classifications depend heavily on the instrument used, and standard cut-offs may not suit our sample [18]. Therefore, we chose to analyze the scores as continuous variables. Further studies should also include other potential influencing factors, such as traumatic childhood experiences, which have already been linked to depression in ACHD [77]. Moreover, future research would benefit from longitudinal designs that allow clearer temporal inferences and the examination of symptom trajectories. Such studies should incorporate functional cardiac indices (e.g., heart rate variability, cardiac output) and treatment status, as both may meaningfully shape the associations observed here. In addition, employing latent-variable approaches (e.g., structural equation modelling) could help reduce construct overlap and yield more precise estimates of the relationships between psychological symptoms and cardiometabolic parameters.

## 5. Conclusions

Overall, our findings provide extremely important insights into key cognitive-emotional characteristics that are central to psychological stress in ACHD. Specifically, our results indicate that variables reflecting a dominant or consuming quality, such as rumination or an engulfing illness identity, as well as emotion regulation strategies such as reappraisal and acceptance, play an important role in the context of depressive and anxiety symptoms in ACHD. The development and further optimization of interventions should particularly address dominant, repetitive thought patterns and preoccupying illness- and self-related concepts. In everyday clinical practice, it appears useful to assess stressful rumination loops to identify vulnerable patients. Elevated rumination and the sense of being strongly preoccupied with the illness should be considered warning signs in ACHD.

## Figures and Tables

**Table 1:** Demographic characteristics of the total sample and for each of the CHD severity levels.

Demographic Characteristic	Total Sample (*N* = 1136)	Simple CHD (*n* = 142)	Moderate CHD (*n* = 618)	Complex CHD (*n* = 376)
**Age in years, *M* (*SD*)**	36.7 (14.0)	34.2 (14.9)	38.5 (14.4)	34.8 (12.6)
**Sex, *n* (%)**				
Male	458 (40.3)	44 (31.0)	264 (42.7)	150 (39.9)
Female	678 (59.7)	98 (69.0)	354 (57.3)	226 (60.1)
**Relationship status, *n* (%)**				
Single	404 (35.6)	50 (35.2)	203 (32.8)	151 (40.2)
In a relationship/married	732 (64.4)	92 (64.8)	415 (67.2)	225 (59.8)
**Number of school years, *M (SD)***	11.37 (1.6)	11.42 (1.3)	11.38 (1.5)	11.35 (1.7)
**Education, *n* (%)**				
Without degree	29 (2.6)	2 (1.4)	15 (2.4)	12 (3.2)
Pupil	14 (1.2)	0 (0.0)	11 (1.8)	3 (0.8)
Elementary school	35 (3.1)	0 (0.0)	19 (3.1)	16 (4.3)
Secondary school	105 (9.2)	15 (10.6)	54 (8.7)	36 (9.6)
Completed apprenticeship	250 (22.0)	26 (18.3)	142 (23.0)	82 (21.8)
Advanced technical college	117 (10.3)	21 (14.8)	59 (9.5)	37 (9.8)
High school diploma	175 (15.4)	23 (16.2)	101 (16.3)	51 (13.6)
University	396 (34.9)	54 (38.0)	210 (34.0)	132 (35.1)
Other	15 (1.3)	1 (0.7)	7 (1.1)	7 (1.9)
**Employment, *n* (%)**				
School	24 (2.1)	2 (1.4)	13 (2.1)	9 (2.4)
Trainee	61 (5.4)	13 (9.2)	23 (3.7)	25 (6.6)
University	129 (11.4)	22 (15.5)	71 (11.5)	36 (9.6)
Part-time job	262 (23.1)	33 (23.2)	132 (21.4)	97 (25.8)
Full-time job	496 (43.7)	60 (42.3)	286 (46.3)	150 (39.9)
Seeking a job	16 (1.4)	2 (1.4)	8 (1.3)	6 (1.6)
Independent	57 (5.0)	7 (4.9)	32 (5.2)	18 (4.8)
Retired	135 (11.9)	10 (7.0)	73 (11.8)	52 (13.8)
Other	55 (4.8)	5 (3.5)	29 (4.7)	21 (5.6)
**Net income, *n* (%)**				
Low (<1750 €)	443 (39.0)	56 (39.4)	225 (36.4)	162 (43.1)
Medium (1750–3.999 €)	555 (48.9)	77 (54.2)	305 (49.4)	173 (46.0)
High (>3999 €)	138 (12.1)	9 (6.3)	88 (14.2)	41 (10.9)
**Size of residence, *n* (%)**				
≤5.000	227 (20.0)	28 (19.7)	111 (18.0)	88 (23.4)
5.001–20.000	307 (27.0)	44 (31.0)	169 (27.3)	94 (25.0)
20.001–100.000	247 (21.7)	30 (21.1)	140 (22.7)	77 (20.5)
100.001–500.000	184 (16.2)	23 (16.2)	101 (16.3)	60 (16.0)
>500.000	171 (15.1)	17 (12.0)	97 (15.7)	57 (15.2)
**Secondary disease(s), *n (%)***	632 (55.6)	66 (46.5)	349 (56.5)	217 (57.7)
**Depressive symptoms (PHQ-9), *n (%)***				
No/minimal symptoms	514 (45.2)	57 (40.1)	280 (45.3)	177 (47.1)
Mild symptoms	372 (32.7)	49 (34.5)	203 (32.8)	120 (31.9)
Moderate symptoms	159 (14.0)	24 (16.9)	79 (12.8)	56 (14.9)
Moderately severe symptoms	64 (5.6)	10 (7.0)	39 (6.3)	15 (4.0)
Severe symptoms	27 (2.4)	2 (1.4)	17 (2.8)	8 (2.1)
**Anxiety symptoms (GAD-7), *n (%)***				
No/minimal symptoms	594 (52.3)	73 (51.4)	329 (53.2)	192 (51.1)
Mild symptoms	378 (33.3)	46 (32.4)	207 (33.5)	125 (33.2)
Moderate symptoms	117 (10.3)	16 (11.3)	54 (8.7)	47 (12.5)
Severe symptoms	47 (4.1)	7 (4.9)	28 (4.5)	12 (3.2)

Note. This table is partly based on data published in our previous work [52], which are reused and expanded here. Variables shown in bold indicate the higher-level variable category, whereas non-bold variables denote the specific category levels.

**Table 3:** Significant predictors of depressive symptoms (PHQ-9) in multiple regression analysis.

Variables	B	Robust SE	ß	T	*p*	95%-CI		Semi-Partial R^2^
						**Lower**	**Upper**	
Constant	3.714	1.763		2.107	0.035	0.255	7.173	
CHD (Reference: complex)								
CHD simple	1.242	0.397	0.081	3.126	0.002	0.462	2.022	0.004
CHD moderate	0.853	0.264	0.083	3.235	0.001	0.335	1.370	0.005
IPQ_Symptoms	0.223	0.081	0.120	2.763	0.006	0.065	0.382	0.004
IIQ_Engulfment	1.555	0.287	0.247	5.421	0.000	0.992	2.117	0.016
IIQ_Enrichment	−0.324	0.139	−0.065	−2.341	0.019	−0.596	−0.052	0.003
HFERST_Rumination	1.646	0.150	0.321	10.978	0.000	1.352	1.940	0.068
HFERST_Reappraisal	−0.890	0.174	−0.149	−5.117	0.000	−1.232	−0.549	0.015
HFERST_Acceptance	−0.840	0.188	−0.137	−4.481	0.000	−1.208	−0.472	0.011
HFERST_Suppression of expression	0.581	0.196	0.098	2.965	0.003	0.196	0.966	0.005

Note. Significance level was set at *p* < 0.025 due to Bonferroni correction. Robust standard errors were calculated using HC4-Method. B = beta. SE = Standard Error. ß = standardized beta. T = B/SE. *p* = significance. CI = Confidence Interval. Semi partial R^2^ = Part correlation squared. Model R^2^ = 0.465, corrected model R^2^ = 0.448.

**Table 4:** Significant predictors of anxiety symptoms (GAD-7) in multiple regression analysis.

Variables	B	SE	ß	T	*p*	95%-CI		Semi-Partial R^2^
						Lower	Upper	
Constant	2.766	1.487		1.860	0.063	−0.152	5.685	
CHD (Reference: complex)								
CHD simple	0.854	0.357	0.065	2.394	0.017	0.154	1.555	0.003
CHD moderate	0.514	0.225	0.059	2.283	0.023	0.072	0.957	0.003
IPQ_Emotional involvement	0.163	0.064	0.112	2.554	0.011	0.038	0.288	0.004
IIQ_Engulfment	1.231	0.261	0.231	4.710	0.000	0.718	1.744	0.014
HFERST_Rumination	1.433	0.128	0.330	11.173	0.000	1.181	1.685	0.072
HFERST_Reappraisal	−0.803	0.152	−0.158	−5.275	0.000	−1.102	−0.504	0.017
HFERST_Acceptance	−1.035	0.159	−0.199	−6.521	0.000	−1.347	−0.724	0.023

Note. Significance level was set at *p* < 0.025 due to Bonferroni correction. Robust standard errors were calculated using HC4-Method. B = beta. SE = Standard Error. ß = standardized beta. T = B/SE. *p* = significance. CI = Confidence Interval. Semi partial R^2^ = Part correlation squared. Model R^2^ = 0.469, corrected model R^2^ = 0.452.

## Data Availability

The original contributions presented in this study are included in the article/Supplementary Material. Further inquiries can be directed to the corresponding author.

## Supplementary Materials

The following supporting information can be downloaded at https://www.mdpi.com/article/10.3390/medsci14010002/s1, Table S1. Predictors of depressive symptoms (PHQ-9) in multiple regression analysis; Table S1a. Predictors of depressive symptoms (PHQ-9) in male ACHD in multiple regression analysis; Table S1b. Predictors of depressive symptoms (PHQ-9) in female ACHD in multiple regression analysis; Table S2. Predictors of anxiety symptoms (GAD-7) in multiple regression analysis; Table S2a. Predictors of anxiety symptoms (GAD-7) in male ACHD multiple regression analysis; Table S2b. Predictors of anxiety symptoms (GAD-7) in female ACHD multiple regression analysis.

## References

[1] Wu W., He J., Shao X. (2020). Incidence and mortality trend of congenital heart disease at the global, regional, and national level, 1990–2017. Medicine.

[2] Baumgartner H., De Backer J., Babu-Narayan S.V., Budts W., Chessa M., Diller G.P., Lung B., Kluin J., Lang I.M., Meijboom F. (2021). 2020 ESC Guidelines for the management of adult congenital heart disease. Eur. Heart J..

[3] Stout K.K., Daniels C.J., Aboulhosn J.A., Bozkurt B., Broberg C.S., Colman J.M., Crumb S.R., Dearani J.A., Fuller S., Gurvitz M. (2019). 2018 AHA/ACC Guideline for the Management of Adults with Congenital Heart Disease: Executive Summary: A Report of the American College of Cardiology/American Heart Association Task Force on Clinical Practice Guidelines. J. Am. Coll. Cardiol..

[4] Liu A., Diller G.P., Moons P., Daniels C.J., Jenkins K.J., Marelli A. (2023). Changing epidemiology of congenital heart disease: Effect on outcomes and quality of care in adults. Nat. Rev. Cardiol..

[5] Shekhar S., Agrawal A., Pampori A., Lak H., Windsor J., Ramakrishna H. (2022). Mortality in Adult Congenital Heart Disease: Analysis of Outcomes and Risk Stratification. J. Cardiothorac. Vasc. Anesth..

[6] Coleman A., Chan A., Zaidi A.N. (2020). The emerging psychosocial profile of the adult congenital heart disease patient. Curr. Opin. Organ Transpl..

[7] Grunwald O., Sakowicz-Hriscu A., Waszkiewicz N., Kozuch M., Dobrzycki S. (2025). Psychiatric and Psychological Implications of Congenital Heart Disease. J. Clin. Med..

[8] Moons P., Van Bulck L., Daelman B., Luyckx K. (2023). Mental health in adult congenital heart disease. Int. J. Cardiol. Congenit. Heart Dis..

[9] Westhoff-Bleck M., Briest J., Fraccarollo D., Hilfiker-Kleiner D., Winter L., Maske U., Busch M.A., Bleich S., Bauersachs J., Kahl K.G. (2016). Mental disorders in adults with congenital heart disease: Unmet needs and impact on quality of life. J. Affect. Disord..

[10] Deng L.X., Khan A.M., Drajpuch D., Fuller S., Ludmir J., Mascio C.E., Partington S.L., Qadeer A., Tobin L., Kovacs A.H. (2016). Prevalence and Correlates of Post-traumatic Stress Disorder in Adults with Congenital Heart Disease. Am. J. Cardiol..

[11] Freiberger A., Richter C., Huber M., Beckmann J., Freilinger S., Kaemmerer H., Ewert P., Kohls N., Henningsen P., Allwang C. (2023). Post-Traumatic Distress in Adults with Congenital Heart Disease: An Under-Recognized Complication?. Am. J. Cardiol..

[12] Stapel B., Winter L., Heitland I., Löffler F., Bauersachs J., Westhoff-Bleck M., Kahl K.G. (2024). Impact of congenital heart disease on personality disorders in adulthood. Eur. J. Prev. Cardiol..

[13] Kovacs A., Bendell K.L., Colman J., Harrison J.L., Oechslin E., Silversides C. (2009). Adults with congenital heart disease: Psychological needs and treatment preferences. Congenit. Heart Dis..

[14] Kovacs A.H., Sears S.F., Saidi A.S. (2005). Biopsychosocial experiences of adults with congenital heart disease: Review of the literature. Am. Heart J..

[15] Kovacs A.H., Luyckx K., Thomet C., Budts W., Enomoto J., Sluman M.A., Lu C.-W., Jackson J.L., Khairy P., Cook S.C. (2024). Anxiety and Depression in Adults with Congenital Heart Disease. J. Am. Coll. Cardiol..

[16] Leezer S., Mehta R., Agarwal A., Saraf S., Messmer M., Phillippi R., Jackson J.L., Roeder M., Marlin A., Peyser N.D. (2024). Patient-Reported Outcomes Among Adults with Congenital Heart Disease in the Congenital Heart Initiative Registry. JAMA Netw. Open.

[17] Lebherz C., Frick M., Panse J., Wienstroer P., Brehmer K., Kerst G., Marx N., Mathiak K., Hövels-Gürich H. (2022). Anxiety and Depression in Adults with Congenital Heart Disease. Front. Pediatr..

[18] Ehmann A.L., Schütte E., Semmler J., Berger F., Bauer U.M.M., Schmitt K., Pfitzer C., Helm P.C. (2025). Enhancing Mental Health in Adults with Congenital Heart Disease: Comparison of Depression and Anxiety Screening Tools. Eur. J. Cardio-Thorac. Surg..

[19] Moons P., Kovacs A.H., Luyckx K., Thomet C., Budts W., Enomoto J., Sluman M.A., Yang H.-L., Jackson J.L., Khairy P. (2018). Patient-reported outcomes in adults with congenital heart disease: Inter-country variation, standard of living and healthcare system factors. Int. J. Cardiol..

[20] Moons P., Luyckx K., Thomet C., Budts W., Enomoto J., Sluman M.A., Lu C.-W., Jackson J.L., Khairy P., Cook S.C. (2021). Physical Functioning, Mental Health, and Quality of Life in Different Congenital Heart Defects: Comparative Analysis in 3538 Patients From 15 Countries. Can. J. Cardiol..

[21] Roseman A., Morton L., Kovacs A.H. (2021). Health anxiety among adults with congenital heart disease. Curr. Opin. Cardiol..

[22] Rassart J., Apers S., Kovacs A.H., Moons P., Thomet C., Budts W., Enomoto J., Sluman M.A., Wang J.K., Jackson J.L. (2017). Illness perceptions in adult congenital heart disease: A multi-center international study. Int. J. Cardiol..

[23] O’Donovan C.E., Painter L., Lowe B., Robinson H., Broadbent E. (2016). The impact of illness perceptions and disease severity on quality of life in congenital heart disease. Cardiol. Young.

[24] Andonian C., Beckmann J., Ewert P., Freilinger S., Kaemmerer H., Oberhoffer-Fritz R., Sack M., Neidenbach R. (2020). Assessment of the Psychological Situation in Adults with Congenital Heart Disease. J. Clin. Med..

[25] Van Bulck L., Goossens E., Apers S., Moons P., Luyckx K. (2021). Illness identity in adults with congenital heart disease: Longitudinal trajectories and associations with patient-reported outcomes and healthcare use. J. Adv. Nurs..

[26] Oris L., Luyckx K., Rassart J., Goubert L., Goossens E., Apers S., Arat S., Vandenberghe J., Westhovens R., Moons P. (2018). Illness Identity in Adults with a Chronic Illness. J. Clin. Psychol. Med. Settings.

[27] Broadbent E., Petrie K.J., Main J., Weinman J. (2006). The Brief Illness Perception Questionnaire. J. Psychosom. Res..

[28] Leventhal H., Steele D. (1984). Illness Representations and Coping with Health Threats. Handbook of Psychology and Health Volume IV Social Psychology Aspects of Health.

[29] Oris L., Rassart J., Prikken S., Verschueren M., Goubert L., Moons P., Berg C.A., Weets I., Luyckx K. (2016). Illness Identity in Adolescents and Emerging Adults with Type 1 Diabetes: Introducing the Illness Identity Questionnaire. Diabetes Care.

[30] Charmaz K. (1995). The body, identity, and self. Sociol. Q..

[31] Ehmann A.-L., Schütte E., Semmler J., Berger F., Bauer U., Schmitt K., Pfitzer C., Helm P. (2025). Mental Health Treatment in Adults with Congenital Heart Disease in Germany: An Online, Cross-Sectional Study of Status, Needs, and Treatment Reasons. J. Cardiovasc. Dev. Dis..

[32] Ehmann A.-L., Pruessner L., Barnow S., Helm P., Bauer U. (2020). Emotion Regulation in Adults with Congenital Heart Disease. Thorac. Cardiovasc. Surg..

[33] Pruessner L., Hartmann S., Ehmann A.-L., Barnow S., Bauer U., Helm P. (2025). Digital Emotion Regulation Interventions for Patients with Congenital Heart Disease: A Randomized Clinical Trial. JAMA Netw. Open.

[34] Chen M.S., Cai Q., Omari D., Sanghvi D.E., Lyu S., Bonanno G.A. (2025). Emotion regulation and mental health across cultures: A systematic review and meta-analysis. Nat. Hum. Behav..

[35] Cludius B., Mennin D., Ehring T. (2020). Emotion regulation as a transdiagnostic process. Emotion.

[36] Wright R.N., Adcock R.A., LaBar K.S. (2025). Learning emotion regulation: An integrative framework. Psychol. Rev..

[37] Sullivan E.C., James E., Henderson L.M., McCall C., Cairney S.A. (2023). The influence of emotion regulation strategies and sleep quality on depression and anxiety. Cortex.

[38] Berking M., Wupperman P. (2012). Emotion regulation and mental health: Recent findings, current challenges, and future directions. Curr. Opin. Psychiatry.

[39] Cserép M., Szabó B., Tóth-Heyn P., Szabo A.J., Szumska I. (2022). The Predictive Role of Cognitive Emotion Regulation of Adolescents with Chronic Disease and Their Parents in Adolescents’ Quality of Life: A Pilot Study. Int. J. Env. Res. Public Health.

[40] Dryman M.T., Heimberg R.G. (2018). Emotion regulation in social anxiety and depression: A systematic review of expressive suppression and cognitive reappraisal. Clin. Psychol. Rev..

[41] Wierenga K.L., Lehto R.H., Given B. (2017). Emotion Regulation in Chronic Disease Populations: An Integrative Review. Res. Theory Nurs. Pract..

[42] Gotlib I.H., Joormann J. (2010). Cognition and depression: Current status and future directions. Annu. Rev. Clin. Psychol..

[43] LeMoult J., Gotlib I.H. (2019). Depression: A cognitive perspective. Clin. Psychol. Rev..

[44] Min J.A., Yu J.J., Lee C.U., Chae J.H. (2013). Cognitive emotion regulation strategies contributing to resilience in patients with depression and/or anxiety disorders. Compr. Psychiatry.

[45] Aldao A., Nolen-Hoeksema S. (2010). Specificity of cognitive emotion regulation strategies: A transdiagnostic examination. Behav. Res. Ther..

[46] Iwakabe S., Nakamura K., Thoma N.C. (2023). Enhancing emotion regulation. Psychother. Res..

[47] Pruessner L., Timm C., Kalmar J., Bents H., Barnow S., Mander J. (2024). Emotion Regulation as a Mechanism of Mindfulness in Individual Cognitive-Behavioral Therapy for Depression and Anxiety Disorders. Depress. Anxiety.

[48] Kazemi Rezaei S.V., Kakabraee K., Hosseini S.S. (2019). The Effectiveness of Emotion Regulation Skill Training Based on Dialectical Behavioral Therapy on Cognitive Emotion Regulation and Quality of Life of Patients with Cardiovascular Diseases. J. Arak Univ. Med. Sci..

[49] Roy B., Riley C., Sinha R. (2018). Emotion regulation moderates the association between chronic stress and cardiovascular disease risk in humans: A cross-sectional study. Stress.

[50] Van Bulck L., Goossens E., Luyckx K., Oris L., Apers S., Moons P. (2018). Illness Identity: A Novel Predictor for Healthcare Use in Adults with Congenital Heart Disease. J. Am. Heart Assoc..

[51] Westhoff-Bleck M., Winter L., Aguirre Davila L., Herrmann-Lingen C., Treptau J., Bauersachs J., Bleich S., Kahl K.G. (2020). Diagnostic evaluation of the hospital depression scale (HADS) and the Beck depression inventory II (BDI-II) in adults with congenital heart disease using a structured clinical interview: Impact of depression severity. Eur. J. Prev. Cardiol..

[52] Ehmann A.-L., Schütte E., Semmler J., Berger F., Bauer U., Schmitt K., Pfitzer C., Helm P. (2025). Key Factors of Adherence in Cardiological Follow-Up of Adults with Congenital Heart Disease. J. Cardiovasc. Dev. Dis..

[53] Helm P.C., Koerten M.A., Abdul-Khaliq H., Baumgartner H., Kececioglu D., Bauer U.M. (2016). Representativeness of the German National Register for Congenital Heart Defects: A clinically oriented analysis. Cardiol. Young.

[54] Jacobs J.P., Franklin R.C.G., Béland M.J., Spicer D.E., Colan S.D., Walters H.L., Bailliard F., Houyel L., St Louis J.D., Lopez L. (2021). Nomenclature for Pediatric and Congenital Cardiac Care: Unification of Clinical and Administrative Nomenclature—The 2021 International Paediatric and Congenital Cardiac Code (IPCCC) and the Eleventh Revision of the International Classification of Diseases (ICD-11). Cardiol. Young.

[55] Kroenke K., Spitzer R.L., Williams J.B. (2001). The PHQ-9: Validity of a brief depression severity measure. J. Gen. Intern. Med..

[56] Williams N. (2014). The GAD-7 questionnaire. Occup. Med..

[57] Izadpanah S., Barnow S., Neubauer A.B., Holl J. (2019). Development and Validation of the Heidelberg Form for Emotion Regulation Strategies (HFERST): Factor Structure, Reliability, and Validity. Assessment.

[58] Warnes C.A., Williams R.G., Bashore T.M., Child J.S., Connolly H.M., Dearani J.A., Del Nido P., Fasules J.W., Graham T.P., Hijazi Z.M. (2008). ACC/AHA 2008 guidelines for the management of adults with congenital heart disease: A report of the American College of Cardiology/American Heart Association Task Force on Practice Guidelines (Writing Committee to Develop Guidelines on the Management of Adults with Congenital Heart Disease). Developed in Collaboration with the American Society of Echocardiography, Heart Rhythm Society, International Society for Adult Congenital Heart Disease, Society for Cardiovascular Angiography and Interventions, and Society of Thoracic Surgeons. J. Am. Coll. Cardiol..

[59] Igo R.P., Salkind N.J. (2010). Influential Data Points. Encyclopedia of Research Design.

[60] Huber P.J. (1981). Robust Statistics.

[61] Figueroa C.A., DeJong H., Mocking R.J.T., Fox E., Rive M.M., Schene A.H., Stein A., Ruhé H.G. (2019). Attentional control, rumination and recurrence of depression. J. Affect. Disord..

[62] Shors T.J., Millon E.M., Chang H.Y., Olson R.L., Alderman B.L. (2017). Do sex differences in rumination explain sex differences in depression?. J. Neurosci. Res..

[63] Moretta T., Messerotti Benvenuti S. (2022). Early indicators of vulnerability to depression: The role of rumination and heart rate variability. J. Affect. Disord..

[64] Chiang Y.H., Ma Y.C., Lin Y.C., Jiang J.L., Wu M.H., Chiang K.C. (2022). The Relationship between Depressive Symptoms, Rumination, and Suicide Ideation in Patients with Depression. Int. J. Env. Res. Public Health.

[65] DeGeorge K.C., Grover M., Streeter G.S. (2022). Generalized Anxiety Disorder and Panic Disorder in Adults. Am. Fam. Physician.

[66] Aldao A., Nolen-Hoeksema S. (2012). The influence of context on the implementation of adaptive emotion regulation strategies. Behav. Res. Ther..

[67] Tyrer P., Baldwin D. (2006). Generalised anxiety disorder. Lancet.

[68] Lamers F., Swendsen J., Cui L., Husky M., Johns J., Zipunnikov V., Merikangas K.R. (2018). Mood reactivity and affective dynamics in mood and anxiety disorders. J. Abnorm. Psychol..

[69] Showraki M., Showraki T., Brown K. (2020). Generalized Anxiety Disorder: Revisited. Psychiatr. Q..

[70] Kotov R., Krueger R.F., Watson D., Cicero D.C., Conway C.C., DeYoung C.G., Eaton N.R., Forbes M.K., Hallquist M.N., Latzman R.D. (2021). The Hierarchical Taxonomy of Psychopathology (HiTOP): A Quantitative Nosology Based on Consensus of Evidence. Annu. Rev. Clin. Psychol..

[71] Ciocca G., Rossi R., Collazzoni A., Gorea F., Vallaj B., Stratta P., Longo L., Limoncin E., Mollaioli D., Gibertoni D. (2020). The impact of attachment styles and defense mechanisms on psychological distress in a non-clinical young adult sample: A path analysis. J. Affect. Disord..

[72] Di Giuseppe M., Lo Buglio G., Cerasti E., Boldrini T., Conversano C., Lingiardi V., Tanzilli A. (2024). Defense mechanisms in individuals with depressive and anxiety symptoms: A network analysis. Front. Psychol..

[73] Smarr K.L., Keefer A.L. (2011). Measures of depression and depressive symptoms: Beck Depression Inventory-II (BDI-II), Center for Epidemiologic Studies Depression Scale (CES-D), Geriatric Depression Scale (GDS), Hospital Anxiety and Depression Scale (HADS), and Patient Health Questionnaire-9 (PHQ-9). Arthritis Care Res..

[74] van der Velden A.M., Scholl J., Elmholdt E.M., Fjorback L.O., Harmer C.J., Lazar S.W., O’Toole M.S., Smallwood J., Roepstorff A., Kuyken W. (2023). Mindfulness Training Changes Brain Dynamics During Depressive Rumination: A Randomized Controlled Trial. Biol. Psychiatry.

[75] Zhou H.X., Chen X., Shen Y.Q., Li L., Chen N.X., Zhu Z.C., Castellanos F.X., Yan C.G. (2020). Rumination and the default mode network: Meta-analysis of brain imaging studies and implications for depression. Neuroimage.

[76] Cladder-Micus M.B., Speckens A.E., Vrijsen J.N., Donders A.R.T., Becker E.S., Spijker J. (2018). Mindfulness-based cognitive therapy for patients with chronic, treatment-resistant depression: A pragmatic randomized controlled trial. Depress. Anxiety.

[77] Stapel B., Löffler F., Westhoff-Bleck M., Heitland I., Kahl K. (2025). Traumatische Kindheitserlebnisse und Herzgesundheit am Beispiel von Erwachsenen mit angeborenem HerzfehlerTraumatic childhood experiences and cardiovascular health using the example of adults with congenital heart disease. Nervenarzt.

