# Supplementary material for: How Emotion Regulation and Illness Identity Shape Mental Health in Adults with Congenital Heart Disease
Source: Med Sci (Basel). 2025 Dec 19;14(1):2. doi: 10.3390/medsci14010002 (PMC12821645; doi:10.3390/medsci14010002)
Supplement: Supplementary file 1 [file medsci-14-00002-s001.zip › medsci-3976187-supplementary.pdf]

SUPPLEMENTARY MATERIAL

Table S1. Predictors of depressive symptoms (PHQ-9) in multiple regression analysis

Variables	B	Robust SE	ß	T	p	95%-CI lower	95%-CI upper	VIF	Semi-partial R^2
Constant	3.714	1.763		2.107	0.035	0.255	7.173		
CHD (Reference: complex)									
CHD simple	1.242	0.397	.081	3.126	0.002	0.462	2.022	1.493	0.004
CHD moderate	.853	0.264	.083	3.235	0.001	0.335	1.370	1.420	0.005
Secondary diseases (yes)	.419	0.247	.041	1.695	0.090	-0.066	0.903	1.184	0.001
Age	.001	0.010	.003	0.109	0.913	-0.019	0.022	1.489	0.000
Sex (female)	.520	0.257	.050	2.019	0.044	0.015	1.024	1.234	0.002
Relationship (yes)	-.082	0.291	-.008	-0.282	0.778	-0.652	0.488	1.348	0.000
Education (Reference: University)									
High school dimploma	.381	0.377	.027	1.012	0.312	-0.358	1.121	1.372	0.001
Advanced technical college	-.091	0.401	-.005	-0.228	0.819	-0.877	0.695	1.238	0.000
Completed Apprenticeship	.100	0.338	.008	0.296	0.768	-0.563	0.763	1.429	0.000
Secondary school	.861	0.512	.049	1.683	0.093	-0.143	1.865	1.332	0.002
Elementary school	-.307	0.697	-.012	-0.440	0.660	-1.674	1.061	1.290	0.000
Pupil	-.339	0.790	-.007	-0.429	0.668	-1.890	1.211	1.113	0.000
Without degree	-1.351	0.735	-.042	-1.838	0.066	-2.793	0.091	1.366	0.001
Net income (Reference: low)									
Medium	-.156	0.284	-.015	-0.548	0.584	-0.712	0.401	1.431	0.000
High	.020	0.375	.001	0.053	0.958	-0.716	0.756	1.453	0.000
City size	.023	0.095	.006	0.242	0.809	-0.163	0.209	1.137	0.000
IPQ_Impairment	-.119	0.082	-.067	-1.445	0.149	-0.281	0.043	3.923	0.001
IPQ_Control	-.041	0.041	-.024	-1.017	0.310	-0.121	0.038	1.117	0.001
IPQ_Treatment Benefit	-.072	0.051	-.037	-1.414	0.158	-0.172	0.028	1.176	0.001
IPQ_Symptoms	.223	0.081	.120	2.763	0.006	0.065	0.382	3.514	0.004
IPQ_Worries	-.085	0.072	-.047	-1.188	0.235	-0.227	0.056	2.694	0.001
IPQ_Understanding	-.084	0.058	-.038	-1.444	0.149	-0.198	0.030	1.228	0.001
IPQ_Emotional involvement	.103	0.071	.060	1.448	0.148	-0.037	0.243	2.839	0.001
IIQ_Engulfment	1.555	0.287	.247	5.421	0.000	0.992	2.117	3.915	0.016
IIQ_Rejection	-.200	0.198	-.031	-1.013	0.311	-0.588	0.188	1.543	0.001
IIQ_Acceptance	.070	0.243	.009	0.289	0.773	-0.407	0.548	1.713	0.000
IIQ_Enrichment	-.324	0.139	-.065	-2.341	0.019	-0.596	-0.052	1.637	0.003
HFERST_Rumination	1.646	0.150	.321	10.978	0.000	1.352	1.940	1.516	0.068
HFERST_Reappraisal	-.890	0.174	-.149	-5.117	0.000	-1.232	-0.549	1.516	0.015
HFERST_Acceptance	-.840	0.188	-.137	-4.481	0.000	-1.208	-0.472	1.748	0.011
HFERST_Problemsolving	-.234	0.208	-.033	-1.121	0.263	-0.642	0.175	1.645	0.001
HFERST_Suppression of expression	.581	0.196	.098	2.965	0.003	0.196	0.966	1.870	0.005
HFERST_Suppression of experience	.235	0.231	.035	1.015	0.310	-0.219	0.688	1.960	0.001
HFERST_Avoidance	.053	0.168	.009	0.317	0.751	-0.277	0.384	1.370	0.000
HFERST_Social sharing	-.266	0.132	-.059	-2.013	0.044	-0.525	-0.007	1.608	0.002

Note. Significance level was set at $p <.025$ due to Bonferroni correction. Robust standard errors were calculated using HC4-Method. B = beta. SE = Standard Error. ß = standardized beta. p = significance. CI = Confidence Interval. VIF = Variance Inflation Factor. Semi partial R^2 = Part correlation squared. Model R^2 = .465, corrected model R^2 = .448.

Table S1a. Predictors of depressive symptoms (PHQ-9) in male ACHD in multiple regression analysis

						95%-CI			
Variables	B	Robust SE	ß	T	p	lower	upper	VIF	Semi-partial R²
Constant	4.218	3.110		1.356	0.176	-1.895	10.331		
CHD (Reference: complex)									
CHD simple	-0.536	0.700	-0.033	-0.766	0.444	-1.912	0.840	1.458	0.001
CHD moderate	0.362	0.433	0.037	0.837	0.403	-0.489	1.214	1.489	0.001
Secondary diseases (yes)	-0.214	0.420	-0.022	-0.511	0.610	-1.039	0.610	1.339	0.000
Age	0.027	0.017	0.086	1.584	0.114	-0.007	0.061	1.689	0.004
Relationship (yes)	-0.210	0.490	-0.021	-0.428	0.669	-1.174	0.754	1.669	0.000
Education (Reference: University)									
High school diploma	0.219	0.593	0.016	0.370	0.712	-0.947	1.385	1.422	0.000
Advanced technical college	0.232	0.675	0.015	0.344	0.731	-1.095	1.560	1.262	0.000
Completed Apprenticeship	0.277	0.567	0.022	0.488	0.626	-0.838	1.391	1.429	0.000
Secondary school	0.907	0.929	0.053	0.977	0.329	-0.918	2.733	1.431	0.002
Elementary school	0.622	1.125	0.030	0.553	0.581	-1.589	2.832	1.408	0.001
Pupil	-0.499	1.401	-0.013	-0.356	0.722	-3.252	2.254	1.178	0.000
Without degree	-0.775	1.216	-0.028	-0.637	0.524	-3.164	1.615	1.369	0.001
Net income (Reference: low)									
Medium	0.024	0.510	0.002	0.047	0.963	-0.978	1.026	1.910	0.000
High	0.322	0.559	0.026	0.576	0.565	-0.777	1.421	1.921	0.000
City size	-0.136	0.144	-0.040	-0.948	0.343	-0.419	0.146	1.153	0.001
IPQ_Impairment	-0.032	0.142	-0.019	-0.228	0.820	-0.312	0.247	3.752	0.000
IPQ_Control	-0.102	0.062	-0.063	-1.650	0.100	-0.223	0.019	1.185	0.003
IPQ_Treatment Benefit	-0.040	0.087	-0.020	-0.461	0.645	-0.212	0.132	1.166	0.000
IPQ_Symptoms	0.231	0.150	0.124	1.538	0.125	-0.064	0.527	3.341	0.005
IPQ_Worries	-0.168	0.128	-0.095	-1.315	0.189	-0.419	0.083	2.620	0.003
IPQ_Understanding	-0.017	0.084	-0.008	-0.200	0.842	-0.182	0.148	1.265	0.000
IPQ_Emotional involvement	0.065	0.135	0.040	0.481	0.631	-0.201	0.331	2.890	0.001
IIQ_Engulfment	1.197	0.472	0.201	2.537	0.012	0.270	2.125	4.172	0.010
IIQ_Rejection	-0.093	0.321	-0.015	-0.289	0.773	-0.724	0.538	1.545	0.000
IIQ_Acceptance	-0.375	0.393	-0.053	-0.954	0.341	-1.147	0.398	1.696	0.002
IIQ_Enrichment	-0.253	0.216	-0.055	-1.175	0.241	-0.677	0.171	1.776	0.002
HFERST_Rumination	1.793	0.235	0.367	7.632	0.000	1.331	2.255	1.344	0.100
HFERST_Reappraisal	-0.777	0.286	-0.134	-2.720	0.007	-1.338	-0.215	1.652	0.011
HFERST_Acceptance	-0.660	0.290	-0.112	-2.275	0.023	-1.231	-0.090	1.730	0.007
HFERST_Problemsolving	-0.368	0.294	-0.058	-1.250	0.212	-0.946	0.211	1.839	0.002
HFERST_Suppression of expression	0.692	0.295	0.117	2.341	0.020	0.111	1.272	1.832	0.007
HFERST_Suppression of experience	-0.031	0.328	-0.005	-0.094	0.925	-0.676	0.614	1.854	0.000
HFERST_Avoidance	0.324	0.232	0.058	1.397	0.163	-0.132	0.780	1.378	0.002
HFERST_Social sharing	-0.274	0.213	-0.060	-1.288	0.199	-0.693	0.144	1.502	0.002

Note. N = 458. Significance level was set at p <.025 due to Bonferroni correction. Robust standard errors were calculated using HC4-Method. B = beta. SE = Standard Error. ß = standardized beta. p = significance. CI = Confidence Interval. VIF = Variance Inflation Factor. Semi partial R^2 = Part correlation squared. Model R^2 = .452, corrected model R^2 = .408.

Table S1b. Predictors of depressive symptoms (PHQ-9) in female ACHD in multiple regression analysis

						95%-CI			
Variables	B	Robust SE	ß	T	p	upper	lower	VIF	Semi-partial R²
Constant	4.179	2.319		1.802	0.072	-0.375	8.733		
CHD (Reference: complex)									
CHD simple	2.197	0.514	0.149	4.271	0.000	1.187	3.207	1.586	0.014
CHD moderate	1.244	0.344	0.120	3.621	0.000	0.569	1.919	1.433	0.010
Secondary diseases (yes)	0.801	0.311	0.076	2.578	0.010	0.191	1.410	1.146	0.005
Age	-0.022	0.014	-0.054	-1.500	0.134	-0.050	0.007	1.546	0.002
Relationship (yes)	-0.074	0.371	-0.007	-0.199	0.842	-0.803	0.655	1.249	0.000
Education (Reference: University)									
High school diploma	0.322	0.522	0.023	0.616	0.538	-0.704	1.347	1.424	0.000
Advanced technical college	-0.309	0.491	-0.018	-0.630	0.529	-1.273	0.655	1.272	0.000
Completed Apprenticeship	-0.115	0.422	-0.009	-0.272	0.786	-0.943	0.714	1.471	0.000
Secondary school	0.764	0.604	0.043	1.265	0.206	-0.422	1.950	1.317	0.001
Elementary school	-1.295	0.991	-0.045	-1.307	0.192	-3.241	0.651	1.292	0.002
Pupil	-0.247	0.839	-0.005	-0.294	0.769	-1.894	1.400	1.110	0.000
Without degree	-2.075	1.196	-0.059	-1.736	0.083	-4.423	0.273	1.478	0.002
Net income (Reference: low)									
Medium	-0.333	0.346	-0.032	-0.961	0.337	-1.013	0.347	1.303	0.001
High	-0.504	0.561	-0.026	-0.898	0.369	-1.605	0.598	1.227	0.001
City size	0.160	0.132	0.040	1.216	0.224	-0.098	0.419	1.150	0.001
IPQ_Impairment	-0.157	0.097	-0.086	-1.611	0.108	-0.348	0.034	4.332	0.002
IPQ_Control	0.006	0.055	0.003	0.107	0.915	-0.102	0.114	1.130	0.000
IPQ_Treatment Benefit	-0.093	0.065	-0.048	-1.417	0.157	-0.221	0.036	1.239	0.002
IPQ_Symptoms	0.208	0.099	0.113	2.100	0.036	0.014	0.403	3.840	0.003
IPQ_Worries	-0.056	0.087	-0.031	-0.643	0.520	-0.228	0.115	2.886	0.000
IPQ_Understanding	-0.143	0.085	-0.062	-1.682	0.093	-0.309	0.024	1.294	0.003
IPQ_Emotional involvement	0.156	0.081	0.089	1.934	0.053	-0.002	0.315	3.007	0.003
IIQ_Engulfment	1.811	0.370	0.282	4.892	0.000	1.084	2.538	3.906	0.020
IIQ_Rejection	-0.151	0.265	-0.023	-0.569	0.570	-0.671	0.370	1.693	0.000
IIQ_Acceptance	0.430	0.331	0.057	1.300	0.194	-0.219	1.079	1.854	0.002
IIQ_Enrichment	-0.405	0.193	-0.079	-2.105	0.036	-0.783	-0.027	1.650	0.004
HFERST_Rumination	1.535	0.201	0.285	7.639	0.000	1.141	1.930	1.620	0.050
HFERST_Reappraisal	-0.887	0.232	-0.148	-3.823	0.000	-1.342	-0.431	1.526	0.014
HFERST_Acceptance	-0.937	0.252	-0.149	-3.719	0.000	-1.432	-0.442	1.819	0.012
HFERST_Problemsolving	-0.173	0.300	-0.023	-0.577	0.564	-0.763	0.416	1.616	0.000
HFERST_Suppression of expression	0.461	0.273	0.078	1.688	0.092	-0.075	0.998	1.913	0.003
HFERST_Suppression of experience	0.285	0.321	0.042	0.887	0.376	-0.346	0.916	2.008	0.001
HFERST_Avoidance	-0.134	0.236	-0.023	-0.565	0.572	-0.598	0.331	1.422	0.000
HFERST_Social sharing	-0.305	0.177	-0.066	-1.722	0.086	-0.652	0.043	1.598	0.003

Note. N = 678. Significance level was set at $p < .025$ due to Bonferroni correction. Robust standard errors were calculated using HC4-Method. B = beta. SE = Standard Error. ß = standardized beta. p = significance. CI = Confidence Interval. VIF = Variance Inflation Factor. Semi partial R^2 = Part correlation squared. Model R^2 = .485, corrected model R^2 = .458.

Table S2. Predictors of anxiety symptoms (GAD-7) in multiple regression analysis

Variables	B	Robust SE	ß	T	p	95%-CI lower	95%-CI upper	VIF	Semi partial R^2
Constant	2.766	1.487		1.860	0.063	-0.152	5.685		
CHD (Reference: complex)									
CHD simple	.854	0.357	.065	2.394	0.017	0.154	1.555	1.493	0.003
CHD moderate	.514	0.225	.059	2.283	0.023	0.072	0.957	1.420	0.002
Secondary diseases (yes)	.032	0.208	.004	0.153	0.878	-0.376	0.440	1.184	0.000
Age	-.009	0.009	-.031	-1.100	0.271	-0.026	0.007	1.489	0.001
Sex (female)	.479	0.218	.054	2.191	0.029	0.050	0.907	1.234	0.002
Relationship (yes)	.140	0.258	.016	0.543	0.588	-0.366	0.646	1.348	0.000
Education (Reference: University)									
High school diploma	.166	0.315	.014	0.529	0.597	-0.451	0.784	1.372	0.000
Advanced technical college	-.277	0.345	-.020	-0.802	0.423	-0.955	0.401	1.238	0.000
Completed Apprenticeship	-.167	0.275	-.016	-0.606	0.544	-0.706	0.373	1.429	0.000
Secondary school	.425	0.431	.028	0.986	0.324	-0.420	1.269	1.332	0.001
Elementary school	.549	0.536	.026	1.024	0.306	-0.503	1.601	1.290	0.001
Pupil	.714	0.761	.018	0.937	0.349	-0.780	2.208	1.113	0.000
Without degree	-.734	0.586	-.027	-1.252	0.211	-1.884	0.416	1.366	0.001
Net income (Reference: low)									
Medium	-.146	0.239	-.017	-0.613	0.540	-0.614	0.322	1.431	0.000
High	.499	0.338	.038	1.477	0.140	-0.164	1.161	1.453	0.001
City size	-.063	0.076	-.019	-0.823	0.410	-0.212	0.087	1.137	0.000
IPQ_Impairment	-.128	0.069	-.085	-1.854	0.064	-0.264	0.008	3.923	0.002
IPQ_Control	-.002	0.034	-.001	-0.063	0.950	-0.068	0.064	1.117	0.000
IPQ_Treatment Benefit	-.080	0.043	-.048	-1.881	0.060	-0.164	0.003	1.176	0.002
IPQ_Symptoms	.122	0.066	.078	1.861	0.063	-0.007	0.251	3.514	0.002
IPQ_Worries	-.043	0.059	-.028	-0.731	0.465	-0.159	0.073	2.694	0.000
IPQ_Understanding	-.023	0.050	-.012	-0.453	0.651	-0.120	0.075	1.228	0.000
IPQ_Emotional involvement	.163	0.064	.112	2.554	0.011	0.038	0.288	2.839	0.004
IIQ_Engulfment	1.231	0.261	.231	4.710	0.000	0.718	1.744	3.915	0.014
IIQ_Rejection	-.016	0.166	-.003	-0.097	0.923	-0.342	0.310	1.543	0.000
IIQ_Acceptance	.152	0.200	.024	0.757	0.450	-0.241	0.545	1.713	0.000
IIQ_Enrichment	-.154	0.114	-.037	-1.357	0.175	-0.378	0.069	1.637	0.001
HFERST_Rumination	1.433	0.128	.330	11.173	0.000	1.181	1.685	1.516	0.072
HFERST_Reappraisal	-.803	0.152	-.158	-5.275	0.000	-1.102	-0.504	1.516	0.017
HFERST_Acceptance	-1.035	0.159	-.199	-6.521	0.000	-1.347	-0.724	1.748	0.023
HFERST_Problemsolving	.040	0.188	.007	0.212	0.832	-0.330	0.410	1.645	0.000
HFERST_Suppression of expression	.262	0.155	.052	1.698	0.090	-0.041	0.566	1.870	0.001
HFERST_Suppression of experience	.326	0.183	.058	1.779	0.076	-0.034	0.686	1.960	0.002
HFERST_Avoidance	-.013	0.141	-.003	-0.094	0.925	-0.291	0.264	1.370	0.000
HFERST_Social sharing	-.117	0.113	-.031	-1.041	0.298	-0.338	0.104	1.608	0.001

Note. Significance level was set at $p < .025$ due to Bonferroni correction. Robust standard errors were calculated using HC4-Method. B = beta. SE = Standard Error. ß = standardized beta. p = significance. CI = Confidence Interval. VIF = Variance Inflation Factor. Semi partial R^2 = Part correlation squared. Model $R^2 = .469$, corrected model $R^2 = .452$.

Table S2a. Predictors of anxiety symptoms (GAD-7) in male ACHD multiple regression analysis

						95%-CI			
Variables	B	Robust SE	ß	T	p	lower	upper	VIF	Semi-partial R²
Constant	1.049	2.394		0.438	0.661	-3.657	5.755		
CHD (Reference: complex)									
CHD simple	0.075	0.560	0.006	0.134	0.893	-1.026	1.177	1.458	0.000
CHD moderate	0.527	0.355	0.067	1.487	0.138	-0.170	1.225	1.489	0.003
Secondary diseases (yes)	-0.279	0.336	-0.036	-0.833	0.406	-0.939	0.380	1.339	0.001
Age	0.008	0.013	0.030	0.580	0.562	-0.018	0.033	1.689	0.001
Relationship (yes)	0.303	0.422	0.038	0.717	0.474	-0.527	1.133	1.669	0.001
Education (Reference: University)									
High school diploma	0.206	0.453	0.019	0.454	0.650	-0.685	1.096	1.422	0.000
Advanced technical college	0.029	0.566	0.002	0.051	0.959	-1.084	1.142	1.262	0.000
Completed Apprenticeship	0.068	0.441	0.007	0.153	0.878	-0.799	0.934	1.429	0.000
Secondary school	0.237	0.715	0.017	0.331	0.741	-1.169	1.642	1.431	0.000
Elementary school	0.445	0.711	0.026	0.626	0.531	-0.952	1.842	1.408	0.000
Pupil	-0.722	0.874	-0.023	-0.826	0.409	-2.439	0.995	1.178	0.000
Without degree	0.373	0.820	0.016	0.454	0.650	-1.240	1.985	1.369	0.000
Net income (Reference: low)									
Medium	-0.550	0.401	-0.070	-1.371	0.171	-1.339	0.239	1.910	0.003
High	0.293	0.506	0.029	0.579	0.563	-0.701	1.287	1.921	0.000
City size	-0.104	0.102	-0.037	-1.016	0.310	-0.305	0.097	1.153	0.001
IPQ_Impairment	-0.099	0.103	-0.072	-0.964	0.336	-0.302	0.103	3.752	0.001
IPQ_Control	-0.029	0.050	-0.022	-0.581	0.562	-0.128	0.070	1.185	0.000
IPQ_Treatment Benefit	-0.005	0.067	-0.003	-0.072	0.943	-0.137	0.127	1.166	0.000
IPQ_Symptoms	0.074	0.102	0.049	0.726	0.468	-0.127	0.275	3.341	0.001
IPQ_Worries	-0.033	0.103	-0.023	-0.321	0.748	-0.236	0.169	2.620	0.000
IPQ_Understanding	-0.058	0.075	-0.034	-0.771	0.441	-0.206	0.090	1.265	0.001
IPQ_Emotional involvement	0.009	0.123	0.006	0.070	0.944	-0.233	0.250	2.890	0.000
IIQ_Engulfment	1.690	0.424	0.349	3.990	0.000	0.858	2.523	4.172	0.029
IIQ_Rejection	0.082	0.241	0.017	0.338	0.735	-0.392	0.555	1.545	0.000
IIQ_Acceptance	-0.013	0.302	-0.002	-0.042	0.967	-0.607	0.582	1.696	0.000
IIQ_Enrichment	-0.204	0.173	-0.054	-1.179	0.239	-0.544	0.136	1.776	0.002
HFERST_Rumination	1.583	0.190	0.397	8.322	0.000	1.209	1.956	1.344	0.117
HFERST_Reappraisal	-0.694	0.228	-0.147	-3.037	0.003	-1.143	-0.245	1.652	0.013
HFERST_Acceptance	-0.660	0.241	-0.137	-2.736	0.006	-1.134	-0.186	1.730	0.011
HFERST_Problemsolving	-0.007	0.255	-0.001	-0.027	0.979	-0.508	0.495	1.839	0.000
HFERST_Suppression of expression	0.201	0.229	0.041	0.876	0.382	-0.250	0.651	1.832	0.001
HFERST_Suppression of experience	0.278	0.280	0.051	0.993	0.321	-0.272	0.828	1.854	0.001
HFERST_Avoidance	-0.012	0.201	-0.003	-0.059	0.953	-0.407	0.384	1.378	0.000
HFERST_Social sharing	-0.098	0.174	-0.026	-0.564	0.573	-0.441	0.244	1.502	0.000

Note. N = 458. Significance level was set at *p* <.025 due to Bonferroni correction. Robust standard errors were calculated using HC4-Method. B = beta. SE = Standard Error. ß = standardized beta. p = significance. CI = Confidence Interval. VIF = Variance Inflation Factor. Semi partial R² = Part correlation squared. Model R² = .470, corrected model R² = .427.

Table S2b. Predictors of anxiety symptoms (GAD-7) in female ACHD multiple regression analysis

Variables	B	Robust SE	ß	T	p	95%-CI lower	95%-CI upper	VIF	Semi-partial R²
Constant	4.061	2.048		1.983	0.048	0.040	8.082		
CHD (Reference: complex)									
CHD simple	1.269	0.479	0.100	2.648	0.008	0.328	2.211	1.586	0.006
CHD moderate	0.533	0.298	0.059	1.789	0.074	-0.052	1.118	1.433	0.002
Secondary diseases (yes)	0.258	0.276	0.028	0.932	0.351	-0.285	0.800	1.146	0.001
Age	-0.022	0.013	-0.063	-1.672	0.095	-0.047	0.004	1.546	0.003
Relationship (yes)	0.099	0.342	0.010	0.291	0.771	-0.571	0.770	1.249	0.000
Education (Reference: University)									
High school diploma	0.127	0.452	0.010	0.281	0.779	-0.760	1.013	1.424	0.000
Advanced technical college	-0.364	0.449	-0.025	-0.811	0.418	-1.247	0.518	1.272	0.000
Completed Apprenticeship	-0.277	0.358	-0.026	-0.773	0.440	-0.980	0.426	1.471	0.000
Secondary school	0.352	0.531	0.023	0.662	0.508	-0.691	1.395	1.317	0.000
Elementary school	0.681	0.895	0.027	0.760	0.447	-1.078	2.439	1.292	0.001
Pupil	2.319	0.967	0.052	2.398	0.017	0.420	4.217	1.110	0.002
Without degree	-2.005	0.920	-0.066	-2.180	0.030	-3.811	-0.199	1.478	0.003
Net income (Reference: low)									
Medium	-0.028	0.300	-0.003	-0.092	0.927	-0.617	0.562	1.303	0.000
High	0.251	0.520	0.015	0.483	0.629	-0.771	1.273	1.227	0.000
City size	-0.012	0.112	-0.003	-0.105	0.917	-0.232	0.209	1.150	0.000
IPQ_Impairment	-0.158	0.098	-0.101	-1.618	0.106	-0.350	0.034	4.332	0.002
IPQ_Control	0.021	0.047	0.014	0.449	0.654	-0.072	0.114	1.130	0.000
IPQ_Treatment Benefit	-0.112	0.055	-0.068	-2.023	0.043	-0.221	-0.003	1.239	0.004
IPQ_Symptoms	0.153	0.090	0.097	1.707	0.088	-0.023	0.330	3.840	0.002
IPQ_Worries	-0.052	0.077	-0.034	-0.676	0.499	-0.205	0.100	2.886	0.000
IPQ_Understanding	-0.028	0.072	-0.014	-0.384	0.701	-0.169	0.114	1.294	0.000
IPQ_Emotional involvement	0.273	0.077	0.180	3.519	0.000	0.120	0.425	3.007	0.011
IIQ_Engulfment	0.948	0.330	0.171	2.868	0.004	0.299	1.597	3.906	0.007
IIQ_Rejection	0.027	0.245	0.005	0.109	0.913	-0.454	0.508	1.693	0.000
IIQ_Acceptance	0.301	0.284	0.046	1.061	0.289	-0.256	0.858	1.854	0.001
IIQ_Enrichment	-0.101	0.164	-0.023	-0.615	0.539	-0.424	0.222	1.650	0.000
HFERST_Rumination	1.340	0.181	0.288	7.399	0.000	0.985	1.696	1.620	0.051
HFERST_Reappraisal	-0.782	0.209	-0.151	-3.748	0.000	-1.191	-0.372	1.526	0.015
HFERST_Acceptance	-1.209	0.223	-0.222	-5.420	0.000	-1.647	-0.771	1.819	0.027
HFERST_Problemsolving	0.013	0.276	0.002	0.048	0.962	-0.529	0.556	1.616	0.000
HFERST_Suppression of expression	0.194	0.210	0.038	0.926	0.355	-0.218	0.606	1.913	0.001
HFERST_Suppression of experience	0.363	0.247	0.061	1.466	0.143	-0.123	0.849	2.008	0.002
HFERST_Avoidance	-0.015	0.198	-0.003	-0.075	0.940	-0.405	0.375	1.422	0.000
HFERST_Social sharing	-0.176	0.152	-0.044	-1.160	0.247	-0.474	0.122	1.598	0.001

Note. N = 678. Significance level was set at *p* <.025 due to Bonferroni correction. Robust standard errors were calculated using HC4-Method. B = beta. SE = Standard Error. ß = standardized beta. p = significance. CI = Confidence Interval. VIF = Variance Inflation Factor. Semi partial R^2 = Part correlation squared. Model R^2 = .473, corrected model R^2 = .445.
